# Fouling Behavior in a High-Rate Anaerobic Submerged Membrane Bioreactor (AnMBR) for Palm Oil Mill Effluent (POME) Treatment

**DOI:** 10.3390/membranes11090649

**Published:** 2021-08-25

**Authors:** Wiparat Chaipetch, Arisa Jaiyu, Panitan Jutaporn, Marc Heran, Watsa Khongnakorn

**Affiliations:** 1 Center of Excellence in Membrane Science and Technology, Department of Civil and Environmental Engineering, Faculty of Engineering, Prince of Songkla University, Songkhla 90110, Thailand; naamtaan001@gmail.com; 2 Expert Center of Innovative Materials, Thailand Institute of Scientific and Technological Research, Khlong Luang 12120, Thailand; arisa@tistr.or.th; 3 Research Center for Environmental and Hazardous Substance Management (EHSM), Department of Environmental Engineering, Faculty of Engineering, Khon Kaen University, Khon Kaen 40002, Thailand; panitju@kku.ac.th; 4 Institut Européen des Membranes, IEM, UMR 5635, CNRS, ENSCM, University of Montpellier, CEDEX 5, 34095 Montpellier, France; marc.heran@umontpellier.fr

**Keywords:** anaerobic membrane bioreactor (AnMBR), wastewater, biofouling, protein, EPS

## Abstract

The characteristics of foulant in the cake layer and bulk suspended solids of a 10 L submerged anaerobic membrane bioreactor (AnMBR) used for treatment of palm oil mill effluent (POME) were investigated in this study. Three different organic loading rates (OLRs) were applied with prolonged sludge retention time throughout a long operation time (270 days). The organic foulant was characterized by biomass concentration and concentration of extracellular polymeric substances (EPS). The thicknesses of the cake layer and foulant were analyzed by confocal laser scanning microscopy and Fourier transform infrared spectroscopy. The membrane morphology and inorganic elements were analyzed by field emission scanning electron microscope coupled with energy dispersive X-ray spectrometer. Roughness of membrane was analyzed by atomic force microscopy. The results showed that the formation and accumulation of protein EPS in the cake layer was the key contributor to most of the fouling. The transmembrane pressure evolution showed that attachment, adsorption, and entrapment of protein EPS occurred in the membrane pores. In addition, the hydrophilic charge of proteins and polysaccharides influenced the adsorption mechanism. The composition of the feed (including hydroxyl group and fatty acid compounds) and microbial metabolic products (protein) significantly affected membrane fouling in the high-rate operation.

## 1. Introduction

Alternative energy sources are widely promoted for sustainable development, including in wastewater treatment. The palm oil industry is one of the industries that can practice effective energy recovery from its waste and wastewater [1,2]. Palm oil mill effluent (POME) has a high potential for energy recovery due to its high chemical oxygen demand (COD), which can be converted to biogas by anaerobic digestion [3,4,5]. POME has high organic content, high organic loading rate (OLR), and high sludge concentration, all of which enhance the potential for methane (CH_4_) production. To produce biogas from POME, anaerobic membrane bioreactors (AnMBRs) have been proposed for their high capacity and small footprint; however, membrane fouling, which causes permeate flux decline, is a substantial limitation of the technique [6,7,8]. An understanding of fouling mechanisms and foulant composition is important to effectively control high-rate AnMBR operation. Many researchers have studied fouling prevention strategies, such as air sparging [9] and filtration mode (relaxation) [10,11], among others. Fouling in a membrane reactor unit can be categorized as either reversible or irreversible. Reversible foulants include biomass, suspended solids, and inorganic precipitates, which form a cake layer attached at the membrane surface. Reversible fouling can be prevented by controlling hydrodynamic conditions or it can be removed by physical cleaning [11,12]. Irreversible foulants, on the other hand, are produced by microbial products, such as extracellular polymeric substances (EPS) forming a gel layer, and soluble microbial products (SMP) accumulating on the cake layer or in the membrane pores [12,13]. Irreversible fouling can be removed by chemical cleaning [11]. A substantial amount of research has evaluated fouling behavior in AnMBR, with different operational conditions affecting cake layer formation, EPS and SMP, and inorganic precipitates [13,14,15,16,17,18]. The operational conditions, especially the OLR, can affect microbial production, biomass concentration, and EPS concentration. The fouling of AnMBR in low-strength wastewater and/or at low-rate loading has been observed in many studies. For example, when an AnMBR was operated for a long term in a low-rate condition, the EPS concentration significantly increased with the increase in OLR [15]. High OLR also induced cake layer formation, which increased transmembrane pressure (TMP) due to high filtration resistance. The addition of biochar to reduce fouling propensity has been proposed as a solution. The addition of biochar resulted in less cake layer formation, as confirmed by confocal laser scanning microscopy (CLSM) and energy diffusive X-ray (EDX) analysis [18]. Under medium to high OLR, EPS accumulation in the cake layer has been shown to contribute most to system fouling, as confirmed by scanning electron microscopy (SEM), EDX, Fourier transform infrared (FTIR) spectroscopy, CLSM, zeta potential, roughness, and contact angle [13]. In addition, a higher OLR can cause EPS to be more viscous and hydrophobic, which makes it adhere easily to the membrane surface [14]. The effects of OLR on fouling have also been confirmed when using a ceramic membrane in AnMBR. During high-loading leachate wastewater treatment, fouling was affected by OLR > mixed liquor suspended solids (MLSS) > EPS > SMP [17].

In this study, a fabricated polysulfone (PSf) hollow fiber membrane was used for POME treatment by AnMBR. The cake layer and bulk suspension were characterized by FTIR, CLSM, roughness, and field emission scanning electron microscopy (FESEM) coupled with energy dispersive X-ray spectrometer (EDS) to observe and evaluate the fouling under high-rate conditions to understand the fouling composition and mechanisms over a long operation period.

## 2. Materials and Methods

### 2.1. Materials

POME and sludge samples were collected from a palm oil factory in Surat Thani, Thailand. The characteristics of POME, including pH, temperature, total COD (TCOD), soluble COD (SCOD), total solids (TS), volatile solids (VS), suspended solids (SS), and volatile suspended solids (VSS), were analyzed after acid fermentation to remove fat, oil, and grease (FOG) and large particles in wastewater (Table 1). The POME had high organic strength with TCOD and SCOD of 242 and 107 g/L, respectively. The TS was 18.5 g/L, while VS was 10.3 g/L. Thus, a large fraction of the solids was volatile. The SS and VSS were 8.9 and 3.2 g/L, respectively.

The inoculum sludge was analyzed following standard methods [19]. The initial concentrations of MLSS and mixed liquor volatile suspended solids (MLVSS) were 32.57 and 26.10 g/L, respectively.

### 2.2. Membrane Production and Characteristics

Polysulfone resin (19 wt%), polyvinylpyrrolidone K30 (2 wt%), and propylene glycol (PEG, 4 wt%) were dissolved in N-Methyl-2-pyrrolidone (NMP) at approximately 70 °C for about 5 h to form a homogeneous dope solution. Then, the dope solution was transferred into a polymer dope tank and kept overnight at 40 °C to eliminate the air bubbles formed during stirring and pouring. The degassed dope solution was used to fabricate polysulfone hollow fiber membranes (PSf) through a dry–wet spinning process. Distilled water and tap water at room temperature were used as bore fluid and coagulant, respectively. The dope solution and bore fluid (water) were pressurized by nitrogen gas through a spinneret, with an outer tube diameter of 1.06 mm and inner tube diameter of 0.66 mm, to from a coagulation bath of water. After separation and solidification, the membranes were collected by a roller. The obtained membranes were immersed in water over 3 days to completely remove the NMP used in the membrane fabrication. Next, the membranes were immersed in 10% aqueous glycerine solution for 1 h to preserve the pore structure during drying. The fabricated membrane had a molecular weight cut-off (MWCO) of 67 kDa. The virgin membranes were characterized for morphology, chemical composition, and roughness by FESEM with EDS (FEI/Apreo, Eindhoven, Netherlands), FTIR (Vertex 70, Bruker, Germany), and atomic force microscope (AFM; Flex Axiom, Nanosurf, Switzerland), respectively.

### 2.3. Experimental Setup and Operation of Anaerobic Membrane Bioreactor (AnMBR)

**Figure 1:**
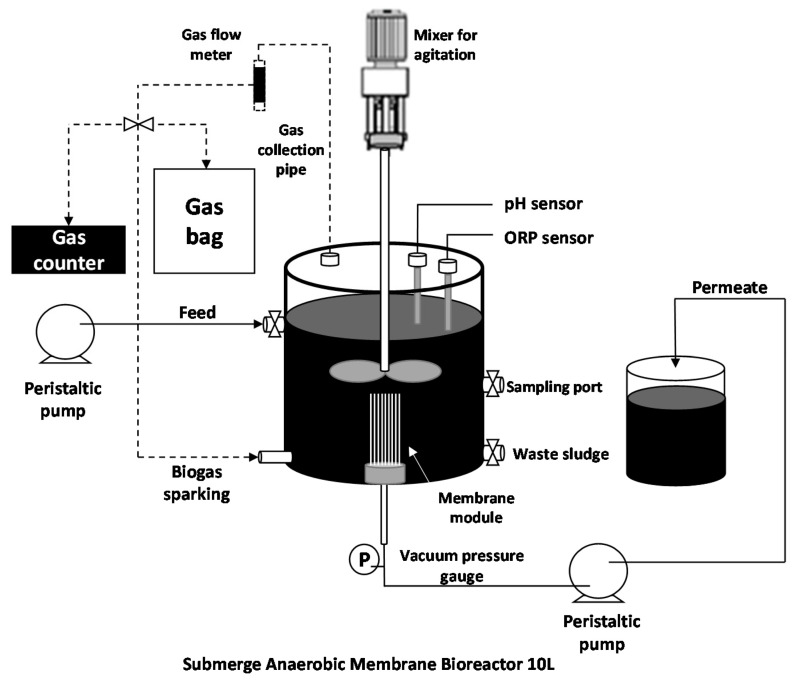
A diagram of the lab-scale submerged anaerobic membrane bioreactor (AnMBR).

A schematic diagram of the AnMBR setup is presented in Figure 1. The AnMBR consisted of a hollow fiber membrane module with total surface area of 0.025 m^2^ in a 10 L reactor. The module was comprised of 65 membrane fibers, each 24 cm in length, and a fiber outside diameter of 0.1 mm. The fibers were potted in a PVC module with epoxy resin, and the module had a diameter of 0.6 cm. The PSf hollow fibers were fixed only at the bottom. The module was operated in an outside-in flow regime under a vacuum pressure in the range of 0.15–0.25 bar, which was supplied by a peristaltic pump (Masterflex, L/S, Cole-Parmer, Chicago, IL, USA). The TMP at the head of the module was measured by the vacuum pressure gauge, which had a similar set-up to a typical membrane bioreactor operation [7,20]. The reactor was operated under a high OLR for 270 days. The overall operation was broken into three 90-day periods, named periods I, II, and III, during which the OLR was modified. The POME feed rate was controlled with a peristatic pump at 3, 4, and 6.7 L/d for periods I, II, and III, respectively, which was equivalent to an OLR of 43, 57, and 99 kg COD/m^3^/d, respectively. At the end of each period, the membrane module was removed and chemically cleaned before the next period. After sludge removal, the fouled membrane from each period was collected for foulant characterization. The physical backwash was set up under 0.5 bar for 1 h followed by the chemical cleaning [11]. The chemical cleaning was achieved by soaking the physically cleaned membrane module in 1% acetic acid solution for 2 h, 1% NaOH for 2 h, and then 10% sodium hypochlorite for 2 h, respectively. The prolonged sludge retention time (SRT) was operated without extraction. However, small samplings were carried out for the purpose of this study.

### 2.4. Biomass Analysis

The biomass sample from the AnMBR system was measured for its MLSS, MLVSS, and EPS concentrations. MLSS and MLVSS in the reactor were measured twice a week using standard methods [19]. EPS solution was extracted from the bulk sludge suspension according to Li et al. [21]. EPS samples were analyzed for protein concentration through a modified Lowry method using a BSA standard [22] and for polysaccharide concentration through the phenol sulfuric acid method with glucose as a standard [23].

### 2.5. Membrane Fouling and Characterization

The membrane filtration was operated under subcritical flux in the TMP constant mode. The permeate flux and permeability rate were measured daily. To minimize physical fouling, gas sparging was added at 1.25 ± 0.25 L/hr through a gas recirculation from the AnMBR tank and internal liquid recirculation. At the end of each period, the foulants in the reactor and at the membrane surfaces were analyzed and characterized, as follows.

- Organic fouling

The hollow fiber membranes in the AnMBR were cut into small pieces of 1 cm length, and the biofilms (attached cells) were dyed with SYTO 9 for 30 min in the dark at room temperature, in order to analyze the distribution of the bacterial cells [24]. Then, the pieces of membrane sample were rinsed with 1 × phosphate buffer saline (PBS) solution to remove excess dye and were incubated for 30 min in the dark, with a mixture of Sypro Orange (green) and Con A Alexa (red). The green represents the total proteins and the red represents polysaccharides, respectively [25]. After that, the membrane samples were rinsed by 1 × PBS solution to remove excess dye from the membrane. The small membrane pieces in the transverse direction (20 mm thick slices) at −20 °C were examined immediately using CLSM (Fluoview FV300/Olympus, Tokyo, Japan). The functional groups of foulants on the membrane surface and the freeze-dried EPS were analyzed with FTIR.

- Inorganic fouling

The membrane preparation was conducted following a procedure reported by Kaya et al. [13]. The membrane morphology and inorganic foulants were characterized by FESEM (FEI/Apreo) coupled with EDS (Oxford). The roughness of virgin and fouled membranes were analyzed by AFM (Flex Axiom, Nanosurf, Switzerland).

## 3. Results

### 3.1. The Relationship of Biomass and EPS

**Figure 2:**
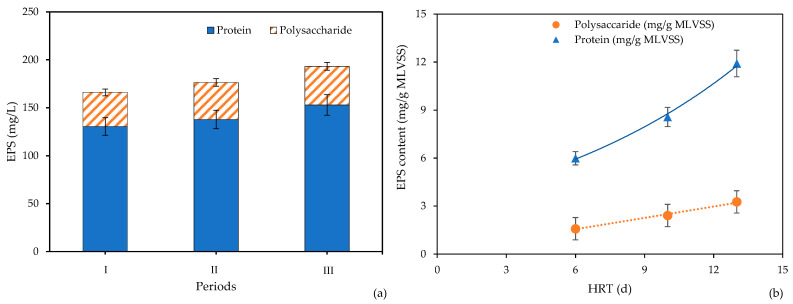
EPS characteristic: (**a**) composition and (**b**) specific EPS content as a function of HRT.

The average of MLSS concentrations during periods I, II, and III were 39.51 ± 2.49, 38.04 ± 1.12, and 36.71 ± 1.17 g/L, respectively. As the OLR in each subsequent period was increased by increasing the feed flow rate, higher concentrations of biomass in the reactor were achieved. In this experiment, a small fraction of biomass was lost as the membrane module was removed from the reactor for chemical cleaning between each period of the experiment. The average EPS concentration was around 166.02, 177.27, and 193.15 mg/L for periods I, II, and III, respectively. Protein made up a large fraction of EPS (77–79%), as shown in Figure 2a. The EPS concentration was not correlated to the biomass concentration, but EPS content increased with the increase in OLR in the reactor. The increase in OLR may have promoted sludge aggregation [26,27]. As shown in Figure 2a, high concentrations of protein were produced rather than polysaccharide. Consistent with this result, a previous study reported that protein had a low first-order kinetics constant (k) compared to polysaccharide. Thus, the increase in SRT can promote greater protein concentration in the biomass, rather than polysaccharide [28]. Biomass-associated product (BAP) formation in the EPS was shown [14,29]. From Table 2 and Figure 2b, the specific EPS was correlated to the average HRT, which agreed well with the results obtained by Santos et al. [29]. The increase in HRT enhanced the degradation of persistent organic substances in high OLR. The ratio of polysaccharine/protein (C/P) ratio was between 0.26–0.28, which was in a similar range to previous studies [21,29]. In addition, the specific protein concentration in EPS increased exponentially, while the polysaccharide increased linearly. A previous study has observed this trend [14]. Increasing feed rate and microorganism concentration caused an increase in C/P. The F/M ratio was higher than 2.5 for high OLR—a finding also confirmed in previous research [30], in which the EPS concentration decreased as F/M decreased. The production of EPS from biological metabolism was conclusively dependent on feed condition, F/M ratio, and HRT.

### 3.2. Membrane Filtration Performance

**Figure 3:**
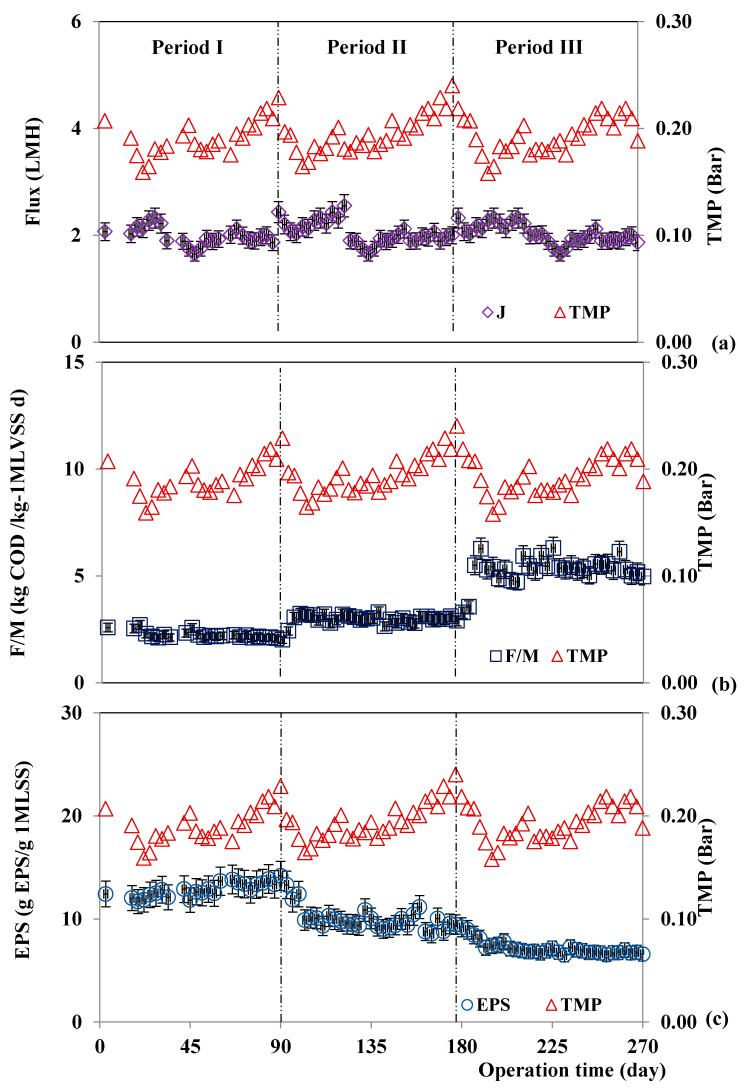
Flux (**a**), F/M (**b**), and EPS (**c**) profiles compared to TMP during operational periods I, II, and III.

The filtration performance was assessed at different OLRs. Meanwhile, the internal recirculation rate with gas sparging was used to inhibit particle accumulation. According to the operational conditions, the average flux was 2.00, 2.04, and 2.02 L/m^2^/h during periods I, II, and III, respectively (Figure 3a). The obtained flux slightly fluctuated at the beginning of the experiment with the controlled TMP lower than 0.3 bar to prevent membrane deformation. Then, the TMP and permeability slightly increased in the middle period and gradually increased until 0.25 bar was reached. The operation of each period was stopped when the final permeate flux was as low as 1.85 L/m^2^/h. Critical flux control and internal recirculation were used to induce shear stresse on the membrane surface to minimize particulate fouling. An accumulation of suspended solids in the reactor increased the viscosity of the supernatant. The high rate of recirculation was intended to avoid clogging and accumulation of solids. The control of hydrodynamic conditions by internal recirculation and the critical flux control can prolong the membrane filtration period, resulting in less frequent cleaning [15,31,32].

OLR increased and HRT decreased from period to period, while TMP rate (dP/dt) was nearly equal (Table 2). Within each period, the TMP increased due to the increase in the resistance of membrane (Figure 3). As TMP increased, the concentration polarization and the number of collisions between particles increased. The increasing pressure also forced the particles to approach the membrane pores, thus inducing pore blocking after cake formation. Also, the EPS accumulation in the cake layer could have been released into the pore, causing pore blocking. High EPS concentration is an indication of biopolymers attached to the membrane surfaces, which also increases membrane resistance [29], especially in period II (Figure 3).

The higher OLR induced an increase in F/M ratio (Figure 3b) and a decrease in specific EPS in the system (Figure 3c). The average F/M ratio was 2.0, 3.2, and 5.5 g COD/g MLVSS/d. The MLSS concentration varied similarly to the F/M ratio and TMP. Once MLSS reached the critical concentration of 40 g/L, it caused TMP to rise rapidly. This jump in TMP was caused by a strongly attached cake layer on the membrane surfaces. On the other hand, for period II, the MLSS concentration did not change, but the TMP jumped at day 159. This result indicated that, not only was TMP affected by MLSS, but also affected by the composition of the colloid or supernatant in the reactor. The TMP result agreed with EPS characterization results (Figure 2), in which the concentration of polysaccharide remained unchanged but proteins increased with time. There is a possibility that the increment of cake layer was related to increasing EPS. The greater protein concentration in the EPS resulted in greater fouling behavior in AnMBR. Many studies have reported that protein, rather that polysaccharide, was the main contributor to membrane fouling [17,18]. In addition, the increase in OLR rather than EPS production significantly affected fouling and was associated with other operational parameters in AnMBR [17]. It should be noted, though, that this study refers to a lab-scale implementation and, therefore, the critical numerical values of MLSS must be verified in pilot- or field-scale plants.

### 3.3. Organic Foulant

CLSM

**Figure 4:**
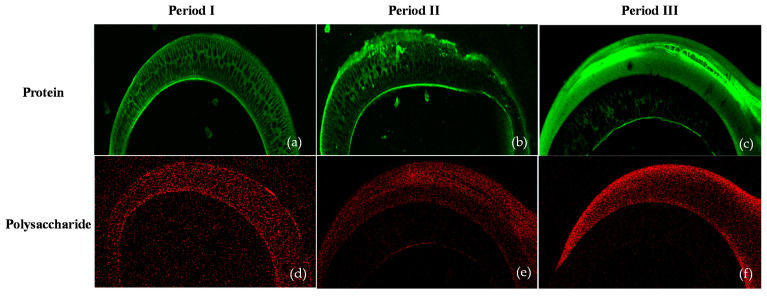
CLSM images of cake layer in AnMBR: period I (**a**,**d**), period II (**b**,**e**), and period III (**c**,**f**).

As seen in Figure 4, CLSM images illustrated the increasing spatial distribution of a thick cake layer and the accumulation of microorganisms and EPS (in the form of protein and polysaccharide). Moreover, as the OLR of the AnMBR reactor increased, the thickness and the specific EPS also increased (Table 2). The thickness of proteins was higher than that of polysaccharides. The spatial distribution of the protein showed that protein more easily attached on the membrane surface than polysaccharide, due to the charge of the membrane surface [17]. At the beginning, our study observed the attachment of a polysaccharide layer followed by the deposition of protein on the cake layer. Matar et al. [25] observed similar results that indicated that protein is a major biofoulant in EPS, as analyzed by CLSM. The distribution of microbial flocs, mainly protein (green color in Figure 4), was clearly found at the bottom of the membrane fibers. It can be concluded that fouling was caused by adsorption of proteins, followed by a deposition of proteins on the membrane surface that leads to the entrapment of proteins in the pores (as can be called pore blockages). The accumulation of protein EPS in the biomass granule and at the membrane surface can occur even under shear force at the surface due to increased gas sparging, demonstrating the accumulation and attachment was caused by the surface charge interaction [33,34]. The presence of proteins and polysaccharides in the hydrophilic fractions of organic substances resulted in irreversible fouling of different membranes.

FTIR

**Figure 5:**
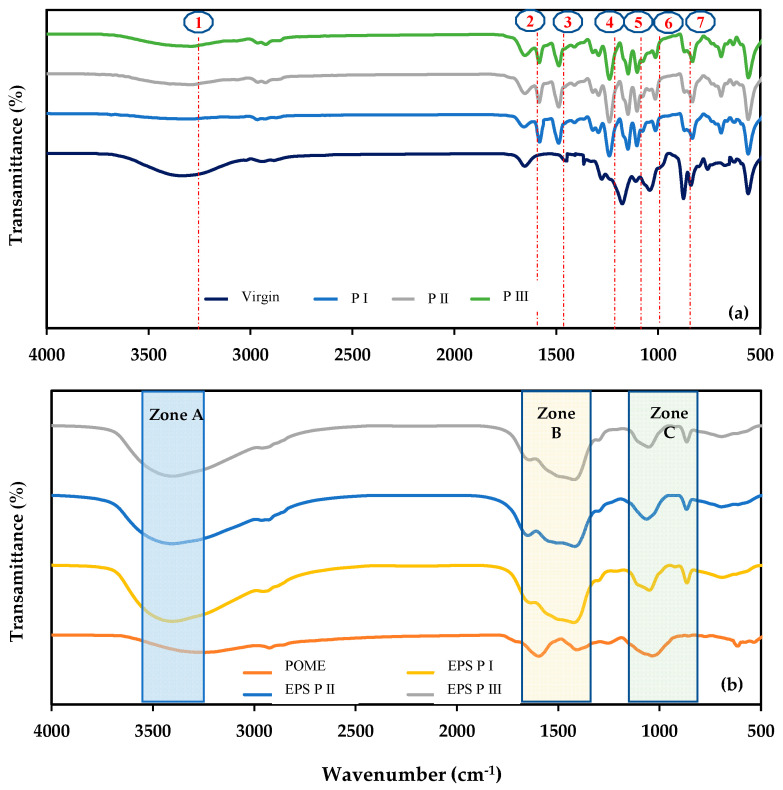
FTIR spectra of (**a**) virgin and fouled membranes and (**b**) POME and EPS in each period.

Summary of FTIR spectra peak assigned for the samples is showed in Table 3. The FTIR spectra of the virgin membrane showed peaks at 3288 cm^−1^, 2964 cm^−1^,1584 cm^−1^, 1244 cm^−1^, 1115 cm^−1^, 832 cm^−1^, and 558 cm^−1^ (Figure 5a). These peaks were attributed to aliphatic amide, O-S-O stretching, C-O-C stretching, C-C aromatic, and C-H stretching of aromatic ring of PSf from the reaction of polymer chain [35]. The fouled membranes had peaks at 1638 cm^−1^ and 1400 cm^−1^ that corresponded to protein EPS in amide I functional group (peak 2) and amide II functional group (peak 3) (Figure 5a), which was also observable in zone B of the EPS (Figure 5b). These peaks were caused by the formation and release of biomass products on membrane surfaces [13]. Strong and high spectra were observed in period II, which correlated to the higher concentration of biomass in the reactor. On the contrary, the high OLR caused a high F/M ratio and induced the excretion of SMP to be higher than EPS, which led to membrane fouling [30]. In addition, the polysaccharides, which contain carbohydrates, presented a peak at 1040 cm^−1^ on fouled membranes. This peak disappeared in EPS but clearly presented in the permeate, suggesting that a fraction of polysaccharides can pass through the membrane. Once the biomass was attached to the membrane surfaces, the cake layer and gel layer were formed, causing biofouling, especially by proteins [25]. The FTIR spectra showed peaks at 3246 cm^−1^, 1597 cm^−1^, 1408 cm^−1^, 1255 cm^−1^, 1036 cm^−1^, and 610–870 cm^−1^ (Figure 5b). These peaks corresponded to hydroxyl ions (O-H stretching), fatty acid and lipids (C-H linkage stretching), aliphatic methylene groups [36], nitrogen compound (C=N stretching), lignin [37], C-O stretching of polysaccharides, phosphorus compounds (P=O stretching), short C chains (humic acids), and inorganics (Si-O complex), respectively [36,37,38]. Moreover, the peak at 1231 cm^−1^ that was assigned to the stretching vibration of P=O in POME disappeared in EPS, but it was observed at low intensity on the fouled membrane surfaces. The absence of a P=O peak has previously been attributed to mineral complexes and phosphorus precipitation on membrane surfaces [39]. The presence of inorganic peaks, silica complexes, and humic acid formation [36] were found at the membrane surfaces, which will be discussed in the inorganic foulant section. Moreover, the peak at 864 cm^−1^ (peak 7), which was related to the stretching vibration of C–O–C from glycosidic bonds, was found in fouled membranes and Zone C of the EPS.

### 3.4. Inorganic Foulant

FESEM

**Figure 6:**
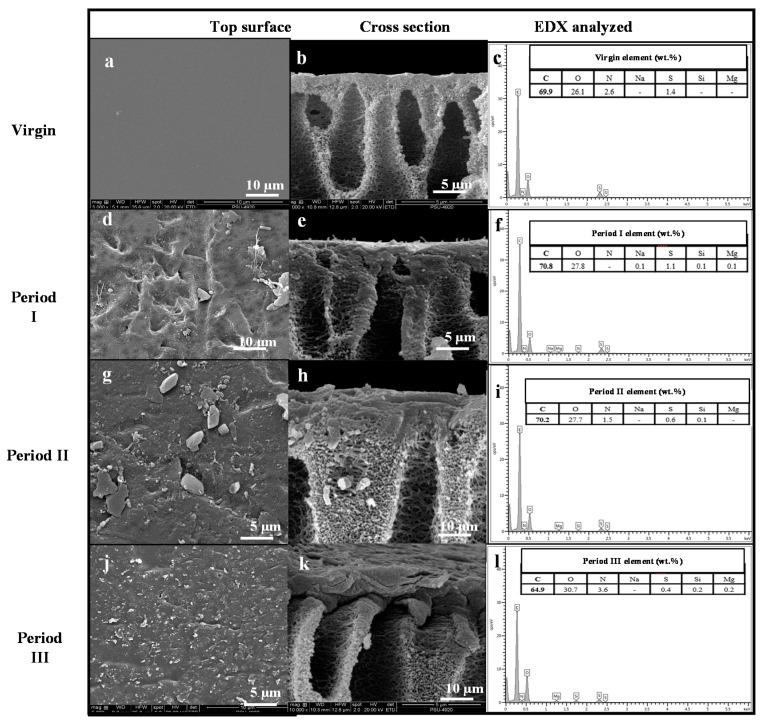
FESEM of the virgin membrane (**a**): top surface, (**b**): cross section, (**c**): EDX element), fouled membrane in period I (**d**): top surface, (**e**): cross section, (**f**): EDX element), fouled membrane in period II (**g**): top surface, (**h**): cross section, (**i**): EDX element), and fouled membrane in period III (**j**): top surface, (**k**): cross section, (**l**): EDX element).

FESEM images of the top surface (Figure 6a) and cross-section (Figure 6b) of the formed cake layer in the ultrafiltration hollow fibers in AnMBR showed that the top surface of the virgin membrane was smooth and uniform, which indicated good fouling resistance of the membrane [25,40]. The elemental composition of the virgin membrane according to EDX was primarily C, O, N, and S (Figure 6c). Differences in the elemental composition of the virgin and fouled ultrafiltration membranes collected from period I (Figure 6f), period II (Figure 6i), and period III (Figure 6l) were observed. The elements Na, Mg, and Si were present in the fouled membranes. The increase in C and O in the fouled membrane implied that the bio-foulant (EPS) covered and interacted with organic compounds on the membrane surfaces [25]. In addition, inorganic scaling on the fouled membranes showed an increasing signal at Mg and Si peaks caused by the increasing OLR. The increased signal at inorganic peaks can be attributed to the evidence presented in FTIR (Figure 5). In addition to bio-foulants, inorganic scaling also induced fouling behavior in the long-term operation of this lab-scale AnMBR used for POME treatment under high OLR. The thickness of the cake layer formed on the membrane surfaces, which was calculated using the constant flux rate, differed according to the OLR. The thickness of the cake layer was 1.067 ± 0.231 µm (Figure 6e), 8.431 ± 0.855 µm (Figure 6h), and 8.366 ± 0.599 µm (Figure 6k), respectively, for the three periods. The cake layer seemed to be caused by the accumulation of microorganisms and their products. Microorganism products, especially the inorganic compounds, interacted with increasing biomass on PSf membrane surfaces. With a MLSS of 40 g/L, the morphology signified the most compact cake layer at the highest OLR in period III (Figure 6k). The foulant layer was comprised of both organic and inorganic substances and a dense and compressed sludge deposition. The morphology and thickness of the foulant layer on the membrane surfaces impacted the filtration performance, as a sudden increase in TMP was observed at the end of each period (Figure 3).

Roughness of the Membrane

**Figure 7:**
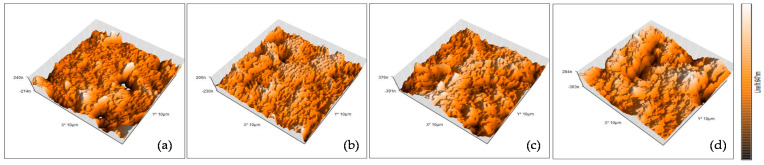
AFM image of the (**a**) virgin membrane, (**b**) fouled membrane in period I, (**c**) fouled membrane in period II, and (**d**) fouled membrane in period III.

The surface probe micrograph (AFM) results indicated a typical morphology (hills and valleys) for membranes (Figure 7). The top surface of the virgin membrane was smooth, which minimized the possibility of solute molecules adhering on the membrane surface and induced less fouling. The average roughness values of the virgin and fouled membranes from period I, II, and III were 44.77, 50.08, 60.58, and 75.80 nm, respectively. The increase in roughness indicated the propensity of pore plugging or fouling of membrane surfaces. The surface of the fouled membranes after the cake layer was removed showed an increase in roughness with the increase in OLR. The EPS components filled the membrane pores as a result of the increase in OLR [41]. The EPS indicated the release of microorganism products that adhered in the membrane pores even though the cake layer was removed. Furthermore, the hydrophilic property of the membrane surface gave it a tendency to interact and form chemical bonds with EPS [7,14]. The membranes with smoothed surfaces were less prone to fouling; therefore, flux decline over time was not observed in this study. Previous studies [7,21,26,41] also reported that the loosely bound EPS had a large effect on the membrane pores for adsorption and deposition of organic and inorganic compounds. In addition, the EPS formation around the biomass granule induced the surface roughness increase, especially at a high loading rate [33,34].

## 4. Discussion

**Figure 8:**
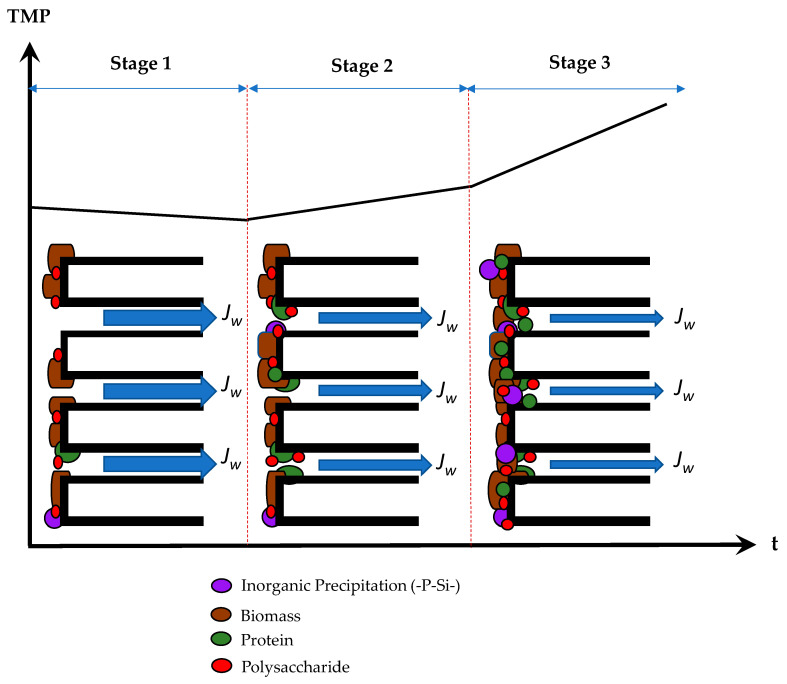
Behavior of foulants in AnMBR for high-rate POME treatment.

This study focused on evaluating fouling mechanisms and characterizing the foulants that occurred on a lab-scale AnMBR used to treat POME. We found that the predominant foulant was protein-EPS, which attached on the membrane surfaces through cake layer formation. The FTIR spectra of the cake layer on the fouled membranes showed peaks at 1638 cm^−^^1^ and 1400 cm^−^^1^. The layer had a compact structure and was high in thickness of the protein portion. The increase of surface roughness reduced the membrane hydrophilicity (O-H stretching). The membrane hydrophobicity caused adhesion of protein molecules, adsorption at the membrane surface, and entrapment within membrane pores, respectively. The pore blocking mechanism occurred because of biofouling accumulation. The accumulation of microorganisms and cake layer increased but EPS products decreased due to the lower HRT. On the other hand, polysaccharides easily detached from the membrane as a result of their hydrophilic properties and the control of hydrodynamic conditions with internal recirculation. In addition, on the membrane surfaces, the cake layer was firmly attached, as was scaling by silica complexes combined with humic acid. The scaling on the fouled membrane was confirmed by EDX and FTIR results. These fouling mechanisms and the behavior of foulants can be considered as a progression (Figure 8). The correlation of fouling phenomena with TMP rising occurred over a 90-day period and could be broken into three stages as follows:

(1) Stage I: initial fouling. The TMP slightly decreased over a short period during days 1 to 20, then a rapid increase of TMP occurred. During the initial decrease of TMP, the flux increased and approached the critical flux (2.8 L/m^2^/h). After that, the cake layer materialized. The flux decreased and reached the local flux instead, causing the TMP to increase from 0.15 bar to 0.18 bar. The organic substances in the bulk feed were the major foulants on membrane surfaces. During this stage, the effect of foulant accumulation in the membrane pores (pore blocking) was minor.
(2) Stage II: intermediate adsorption fouling. From day 20 to 70, the TMP remained constant at 0.20 bar. The cake layer was attached to the membrane surfaces, for which EPS, especially proteins, was adsorbed on the surfaces. A fraction of EPS was accumulated in the membrane pores and was entrapped there by the charge adsorption process.
(3) Stage III: cake and pore blocking. Day 71 onwards, the TMP had risen to 0.25 bar. A dense cake layer accumulated at the surface, and pore blocking occurred simultaneously. In addition, adjacent to the cake layer, the bound EPS released and attached to the membrane.

Overall, this study presented insight into the effect of operational parameters on fouling behavior in the lab-scale AnMBR during long-term operation. However, in order to commercialize AnMBR for POME treatment in a full-scale operation, the reported design and management results obtained need to be verified on larger-scale systems, and pre-treatment to remove suspended solid may be needed to mitigate membrane fouling.

## 5. Conclusions

A high organic loading rate anaerobic membrane bioreactor was operated for 270 days (considered a long operation period). Fouling mechanisms were investigated, providing the following conclusion:

- The growing cake layer, which resulted from high OLR and high MLSS, initiated biofilm formation on the membrane surfaces. The biofilm, in turn, bridged across the pores, resulting in increased TMP.
- EPS accumulated on the cake layer and, thus, plugged the membrane pores. The fouling from polysaccharide EPS can be mitigated by control of hydrodynamic conditions using internal recirculation. Due to the charge on the surfaces and the interaction between proteins and the membrane surface, the removal of protein EPS fouling was more difficult.
- The precipitation of inorganic compounds, silica, and phosphorus, also occurred in the AnMBR system. These compounds were then attached to the cake layer and caused membrane fouling.

Hence, in order for the lab-scale AnMBR to extend its operation time with high OLR, the two following conditions must occur: MLSS must be lower than 40 g/L and the internal recirculation must be higher than 1.25 L/d. High internal recirculation is needed to create violent hydrodynamic turbulence and minimize fouling. Furthermore, to improve the surface charge of the membrane (i.e., for fouling mitigation), future research should focus on the modification of membrane surface properties to reduce the charge interaction between foulants and membrane surfaces.

## Figures and Tables

**Table 1:** Characteristics of palm oil mill effluent (POME) after pretreatment.

Parameter	pH	Temp.	TCOD	SCOD	TS	VS	SS	VSS
		(°C)	(g/L)	(g/L)	(g/L)	(g/L)	(g/L)	(g/L)
Pretreated POME	5.11	35	242	107	18.5	10.3	8.9	3.2

Note: total chemical oxygen demand (TCOD), soluble COD (SCOD), total solids (TS), volatile solids (VS), suspended solids (SS), and volatile suspended solids (VSS).

**Table 2:** EPS substances and fouling parameters evaluated for each OLR.

Period	OLR (kgCOD/m^3^ × d)	HRT (d)	EPS (mg/g MLVSS)			CLSM Thickness (µm)		TMP Rate (bar/d)
			Polysaccharide	Protein	C/P	Polysaccharide	Protein	
I	43	13	3.26 ± 0.13	11.91 ± 0.77	0.27	10.16 ± 2.33	10.74 ± 2.91	0.191 ± 0.017
II	57	10	2.41 ± 0.25	8.57 ± 0.94	0.28	15.34 ± 2.97	17.52 ± 3.99	0.195 ± 0.017
III	99	6	1.58 ± 0.17	5.99 ± 0.66	0.26	16.24 ± 2.74	157.56 ± 81.46	0.192 ± 0.016

**Table 3:** Summary of FTIR spectra peak assigned for the samples.

Sample	Wavelength (cm^−1^)
POME	3246, 1597, 1408, 1255, 1036, 610–870
Virgin membrane	3288, 2964, 1584, 1244, 1115, 832, 558
Fouled membrane	1638, 1400, 1231, 1040, 864
EPS	3285, 1638, 1400, 1231, 1040, 864

## Data Availability

Not applicable.
